# Introducing Single Dose Liposomal Amphotericin B for the Treatment of Visceral Leishmaniasis in Rural Bangladesh: Feasibility and Acceptance to Patients and Health Staff

**DOI:** 10.1155/2014/676817

**Published:** 2014-01-21

**Authors:** Eva-Maria Maintz, Mahbub Hassan, M. Mamun Huda, Debashis Ghosh, Md. Shakhawat Hossain, Abdul Alim, Axel Kroeger, Byron Arana, Dinesh Mondal

**Affiliations:** ^1^Faculty of Medicine, Albert Ludwigs University, 79085 Freiburg, Germany; ^2^International Centre for Diarrhoeal Diseases Research, Bangladesh (ICDDR,B), Dhaka 1212, Bangladesh; ^3^Liverpool School of Tropical Medicine, Liverpool L3 5QA, UK; ^4^Special Programme for Research and Training in Tropical Diseases (TDR-WHO), 1211 Geneva, Switzerland

## Abstract

*Background.* For the treatment of visceral leishmaniasis in Bangladesh, single dose liposomal amphotericin B (ambisome) is supposed to be the safest and most effective treatment. Specific needs for application and storage raise questions about feasibility of its implementation and acceptance by patients and health staff. *Methods.* The study was carried out in the most endemic district of Bangladesh. Study population includes patients treated with ambisome or miltefosine, hospital staff, and a director of the national visceral leishmaniasis program. Study methods include direct observation (subdistrict hospitals), open interviews (heath staff and program personnel), structured questionnaires, and focus group discussions (patients). *Results.* Politicalcommitment for ambisome is strong; the general hospital infrastructure favours implementation but further strengthening is required, particularly for drug storage below 25°C (refrigerators), back-up energy (fuel for generators), and supplies for ambisome administration (like 5% dextrose solution). Ambisome created high satisfaction in patients and hospital staff, less adverse events, and less income loss for patients compared to miltefosine. *Conclusions.* High political commitment, general capacities of subdistrict hospitals, and high acceptability favour the implementation of ambisome treatment in Bangladesh. However, strengthening of the infrastructure and uninterrupted supplies of essential accessories is mandatory before introducing sLAB in Bangladesh.

## 1. Background

Visceral leishmaniasis (VL), also called Kala-azar (KA), is a vector borne neglected disease ranked by the WHO as the infectious disease with the ninth highest burden worldwide [1]. More than 70 per cent of the cases worldwide occur in India, Bangladesh, and Nepal [2]. The annual incidence for Bangladesh has recently been estimated to be between 12,400 and 24,900 cases [2]. Out of the 64 districts and 493 subdistricts of Bangladesh, 45 districts and 105 subdistricts are affected [3].

*L. donovani* causing VL in the region has no other reservoir than humans and no other vector other than *Phlebotomus argentipes*. There are new rapid diagnostic tests [4] and different treatment options so that the elimination of the disease was envisaged. In 2005 a Memorandum of Understanding was signed by Bangladesh, India, and Nepal to eliminate VL from the Indian subcontinent aiming for less than one case per 10,000 people in the endemic districts by 2015 [5].

One important element in the elimination strategy is early diagnosis and complete treatment. Within the last decade new drugs have been developed like paromomycin, miltefosine (hexadecylphosphocholine), and liposomal amphotericin B (LAB), providing additional treatment options to SSG (sodium stibogluconate) and amphotericin B. Recent studies in India show that a single intravenous infusion of liposomal amphotericin B (sLAB) at 10 mg/kg is highly effective in India [6] which is a great improvement over the 28-day oral application of miltefosine or long term injections of paromomycin. It has been suggested by the WHO Regional Technical Advisory Group [7] and the WHO Advisory Panel for Leishmaniasis Control [1] to use sLAB as the first line drug for the VL elimination program in the Indian subcontinent. In 2010 a WHO arrangement with Gilead Sciences Inc., producing ambisome, provided a donation of over 450,000 doses of ambisome over the next 5 years for the treatment of VL patients in Bangladesh, Sudan (North and South), and Ethiopia. Ambisome is currently the only formulation of LAB registered by the United States Food and Drug Administration (US-FDA) and in Bangladesh for the treatment of VL. However, one important difficulty in the application of ambisome is that it requires trained staff, the equipment for IV infusion, testing of haemoglobin, and other clinical parameters as well as special storage requirements ensuring that the drug is not exposed to temperatures above 25°C.

In Bangladesh, the treatment of almost all VL patients is carried out through the public sector, treating patients at subdistrict hospitals called Upazila Health Complexes (UHCs). Bangladesh is the first country aiming at introducing sLAB at the Primary Health Care level for the treatment of VL patients.

Currently icddr,b (International Centre for Diarrhoeal Disease Research, Bangladesh) in collaboration with the Directorate General of Health Services, Government of Bangladesh, and TDR-WHO (Special Programme for Research and Training in Tropical Disease at the World Health Organization), Geneva has been conducting an open trial with single dose ambisome treatment for VL to determine the feasibility of implementing the drug for the treatment of VL at the subdistrict hospital level in Bangladesh. As a part of this study we assessed separately whether the subdistrict hospitals have the necessary facilities to implement ambisome, its acceptance by the hospital staff and patients who have been treated in the above mentioned trial, and compared prospects and limitations of sLAB regimen with the miltefosine treatment which currently is the first line drug for the treatment of VL in Bangladesh, India, and Nepal.

## 2. Methods

### 2.1. Study Sites, Population, and Sampling

The study was conducted in Mymensingh, the most VL endemic district in Bangladesh; within Mymensingh the five most VL affected subdistricts (upazilas) were included: Fulbaria, Trishal, Bhaluka, Muktagacha, and Gaffogaon [8]. The study included rural hospitals, UHCs in these five subdistricts, VL program personnel at the UHC level and central level, and VL patients treated with sLAB regimen or miltefosine. All patients treated with single dose ambisome in the above mentioned ongoing open trial were interviewed directly and one month after treatment. VL patients treated with miltefosine were identified through subdistrict hospital registers. Sample size considerations for the comparison between sLAB and miltefosine treatment regimens are as follows: after estimating the proportion of sLAB patients with side effects (1.3% of vomiting within one month of treatment) and of miltefosine patients (36% of vomiting as reported by doctors) a sample size of 25 patients was calculated to reach a significant difference in the most concerning side effect for miltefosine (defining a power of 80% and a significance level of 5%).

### 2.2. Methods

The study used the following mixed methods of quantitative and qualitative data collection.

- Semistructured interviews, using a list of guiding questions, were conducted with the hospital staff (5 hospital directors, 5 medical officers for disease control, 7 nurses, and 10 technicians). Interviews with hospital head and medical staff were recorded digitally for further clarification; answers by nurses and technician were added to the checklist assessment in written form. Doctors were asked about their preference about the existing treatment regimen for VL.
- A checklist for supplies required for the treatment with ambisome was established, following the standard operation procedure (SOP) for ambisome treatment used in the efficacy trial.
- Using the checklist direct observation of the facilities in the UHC, where the eventual ambisome program will be started, was performed.
- Interviews with VL patients treated with sLAB or miltefosine were conducted using a structured questionnaire. Information was collected about their knowledge, attitude, and practice for VL; their satisfaction about the treatment received; and their preference for sLAB or miltefosine.

Two focus group discussions (FGDs) with VL patients treated with miltefosine were performed using a qualitative method to gain a deeper insight into the general VL management practise as miltefosine patients represent the population treated at the UHC under normal and no trial situation as for sLAB patients.

### 2.3. Data Analysis

Data were entered using EPI Info (version 3.5.1). For data quality assurance, range and consistency checks were performed. SPSS software (version 17.0) was used for data analysis. Descriptive summary statistics including mean, median, standard deviation, and range were explored. Comparison between means or medians was performed by using *t*-test or equivalent nonparametric test where applicable.

Chi-square or Fisher test was used to compare proportions of different groups as required. A two-tailed *P* value ≤ 0.05 was considered significant. Open questions were coded inductively. Qualitative data were analysed in the following way: interviews and the FGDs were recorded and transcribed into English as needed and content analysis was performed. Themes of questionnaires were triangulated with the answers and grounded theory was used analysing additionally emerging themes or new problems described [9]. Transcripts were grouped and categorized into themes that were treated in the questionnaires and new upcoming topics were tabulated using Excel 2010.

## 3. Results

### 3.1. Opinions of Health Staff and Direct Observation of Hospital Facilities

The five directors (Upazila Health and Family Planning Officers (UH&FPO)) interviewed in the rural hospitals (UHCs) of the five subdistricts (upazilas) informed us that in each hospital there were 51 beds (at 3 of the 5 UHCs) or that they were in the process of stocking up from 31 to 51 beds (at 2 of the 5 UHCs). The monthly bed occupancy rate ranged from 70% to over 100% with a mean of 88.3%. On average each month 23.3 VL patients (range from 10 to 40) were treated at each UHC. In each UHC the average number of doctors and nurses involved in VL patients management was 1.8 (range, 1 to 2) and 2.4 (range, 2 to 4), respectively. Except for 1 UHC all directors reported that two nurses at their UHCs had received training for the preparation and administration of single dose ambisome that had happened on average 1.5 years ago.

According to the directors the diagnosis and treatment of VL patients were currently provided at no cost for VL patients. However, according to the laboratory technicians, the uninterrupted supply of rK39 rapid tests (the most important laboratory tool for the diagnosis of VL) was available in 3 of 5 hospitals. Haemoglobin testing facilities were available at all hospitals.

The study team observed a total of 27 refrigerators in the five hospitals; 12 were not functioning. Ice packs and ice boxes were available in all hospitals. Refrigerators for maintaining 4°C to 25°C for drug storage were not available in all hospitals. Each UHC had a back-up generator for power during power cuts. Directors reported average needs of 81.6 L of fuel per month to bridge power cuts and an average amount of 57.5 L of fuel to each UHC per month provided by the central management.

The essential supplies needed for the implementation of single dose ambisome in the five UHCs (see Table 1) were not available in all of the hospitals. However the availability of drugs for management of adverse events during treatment with single dose ambisome was better (Table 2).

### 3.2. Experiences and Opinions of Medical Professionals regarding the Feasibility of Single Dose Ambisome Treatment

Five medical officers, seven nurses, and ten medical laboratory technicians (five technicians of vaccine management and five technicians of the pathology department) participated in the study. All five doctors had treated VL patients on average for 1.6 years; they all had experiences with miltefosine treatment, 4 out of 5 with antimonials (SSG), 1 out of 5 with paromomycin, and 1 doctor with single dose ambisome. All 5 doctors had heard about the sLAB treatment regimen. Four of them thought that sLAB application would be possible at the rural hospital if essential logistics and supplies were available. They felt the staff had sufficient time to handle the work of preparing and applying single dose ambisome. Nurses in the wards had similar views. The doctors mentioned that “*VL patient numbers are getting less*” and “*some nurses have already been trained*.” Two of the doctors had received training on ambisome treatment. Three doctors mentioned difficulties mainly because of the price of ambisome and availability issues for the drug; one mentioned power disruption (“load shedding”) as an issue and the need for storage facilities and refrigerators. Others mentioned the need for trained staff. 4 of the 5 doctors thought that sLAB was the best option for VL treatment.

### 3.3. Views of the Deputy Program Manager of the National Kala-Azar Control Program regarding the Feasibility of Single Dose Ambisome Treatment in Rural Hospitals of Bangladesh

The national director of Kala-azar control (Directorate General of Health Services, Government of Bangladesh) considers sLAB a highly effective treatment that required support particularly during the implementation phase. He informed us that the national program was currently improving knowledge and skills of human resources and building institutional capacities. According to the director, in the past the WHO funded training programs (96 doctors and 96 nurses). The Director stated that a training program, for two doctors and two nurses from 105 UHCs of 105 VL endemic sub districts (upazilas), will be funded by the Government of Bangladesh. The Director was well informed about the requirements for implementing the single dose ambisome program in rural hospitals of VL endemic areas. He mentioned the fuel for generators, the need for more refrigerators, essential supplies for ambisome administration, drugs for management of adverse effects related to ambisome administration, and training for hospital staff. He also mentioned that for the supplies for administering single dose ambisome additional funds might be needed. He was aware that for distribution of ambisome, drugs, diagnostics, and other supplies will need to be organised from the central level and mentioned that it will be done using microplanning and checklists to monitor and compare patients treated and supplies needed for each month. The director hoped that in the next five years, during the donation phase of ambisome, a substantial decline of VL cases can be achieved and that after these five years the national program can afford to continue sLAB treatment free of cost for the patients due to less occurring VL cases.

### 3.4. Patients' Experiences and Perceived Benefits of Miltefosine versus sLAB Treatment

Disappearance of fever, the cardinal symptom of VL, is the first clinical criterion for treatment efficacy. The mean time for fever reduction was 2.5 (SD 0.9) days and 17.6 (SD 11.3) days, *P* < 0.001, for sLAB and miltefosine, respectively (Table 3). During or after treatment VL patients may experience nausea, vomiting, diarrhoea, and abdominal pain. Patients who had been treated with sLAB reported these symptoms less frequently compared to miltefosine patients after one month (Table 3). Time to feel better (recovery) and to start work again was significantly less among the group treated with sLAB compared to those treated with miltefosine (Table 3). Using an average income of a VL household member in Mymensingh of 0.3$, estimated by Anoopa Sharma et al. in 2006 [10], the average income loss due to recovery time was 2.2 USD and 15.85 USD, respectively, for sLAB and miltefosine group. Finally, all patients interviewed indicated in the interviews that they would suggest sLAB treatment to other VL patients whereas the recommendation for miltefosine was given by only 45.5% of patients treated with miltefosine. Likewise, in the focus group discussions miltefosine treated patients were very critical about their treatment and the majority would prefer a one saline treatment staying one night in hospital.

## 4. Discussion

The introduction of a new drug into resource poor settings requires special considerations and precautions. Examples are the introduction of ACT for malaria treatment [11] and TB drugs in the developing world [12]. Likewise, the introduction of miltefosine, the first oral drug against VL, required intensive testing and operational research to be accepted as a first line drug in Bangladesh, India, and Nepal [13].

Special requirements for introducing a new drug or treatment scheme in a resource poor setting include the following.

- The drug should be cost effective and high drug costs would be prohibitive.
- Logistics for drug distribution should be simple: distribution and storage should require little resources.
- Drug management should be simple and the application of the drug should require little skill.
- Monitoring should be straightforward particularly regarding the distribution and drug-related side effects as well as compliance to the treatment.

Most of these requirements are better met by sLAB than by miltefosine or the other VL drugs. There are, however, along with the positive factors some drawbacks to sLAB treatment, discussed below.

### 4.1. Preparing the Large Scale Introduction of sLAB as First Line Treatment in Bangladesh

Enabling factors for the introduction of sLAB in Bangladesh were highlighted in this paper; they include the following.

- *Political commitment* for the implementation of single dose ambisome as first line VL treatment in Bangladesh is high: SOPs are being developed, staff training is taking place, and missing supplies will be distributed from central level using microplanning.
- *WHO support*: first training units for health staff have been financed by TDR-WHO and have now been handed over to the government. The drug donation (see below) has been facilitated by WHO; the clinical community trial has been supported by WHO.
- *Donation by the drug company* (Gilead): over 445 000 vials will be donated over the coming 5 years; this has eliminated one of the major obstacles, which were the costs of the product.
- *Support by research institutions (particularly icddr,b)*: the work since 2005 in relation to active case detection, case management, and drug efficacy has been an important contribution to paving the way for better first line treatments.
- *Contributions of an NGO (MSF)*: through providing a multiple dose of sLAB to 1439 VL patients (15 mg in 3 doses over 5 days) over a period of 25 months, the safety and efficacy of LAB could be underlined: the initial cure rates were 99.6% and 2.7% relapses (which occurred mainly 6 months after treatment), [14]. Furthermore, the MSF experiences showed that trained health workers were able to “independently prepare and administer the appropriate dose” and refer severe cases to the district hospital [14].
- *Feasibility according to our study*: the general capacities of the Upazila Health facilities regarding the expected bed occupancy rate, expected case load of VL patients per month, and patient : physician and patient : nurse ratios were sufficient to perform sLAB treatment at primary care level including the hospitalization of VL patients for two days.
- *Acceptance of sLAB treatment* (compared to miltefosine treatment) by patients *in our study*: (a) sLAB treatment was well tolerated showing only mild side effects (short fever peak after infusion (80.94%) and short duration) which were quickly forgotten by the patients (documented by interviews one month later). (b) sLAB treated patients showed very fast recovery. (c) sLAB patients were satisfied and all patients would recommend the treatment to others and prefer a single infusion to tablets over 28 days. (A certain level of bias has to be considered in the patient interviews as the sLAB patients were interviewed in a trial situation where many particular patient needs could be met, which was not the case for miltefosine treatment.) However, the overall positive response of sLAB patients is in accordance with all other objective and subjective assessments.
- *Other advantages of sLAB over miltefosine*: (a) a single dose treatment ensures 100% adherence rates and its use is safe during pregnancy [15], which are clear advantages over miltefosine where adherence issues have been reported [16] and where pregnancy testing and contraception during treatment are required but rarely performed in reality [17]. (b) The fast recovery (time to be able to work) of sLAB patients with 2.72 days compared to 52.82 days in miltefosine patients reduces the income loss of the patients due to the treatment by about 15$ which is a substantial amount for poor people [18].
- The preliminary *assessment by doctors* was very positive, describing sLAB as the best treatment option if refrigerators would be provided.

However, according to our findings there are certain requirements to be met when introducing sLAB treatment in rural Bangladesh. These include the following.

- *Cold chain issues* are now less stringent for ambisome as it can be stored below 25°C but in tropical temperatures refrigeration is still needed; refrigerators, preferably ice lined refrigerators (ILRs), to bridge power cuts should be provided to ensure the stability of the drug.
- Clinical *monitoring tools* like scales are needed and their quality has to be assured. The majority of *supplies required for ambisome administration* are not yet available at all rural hospitals. Even when distributed from central level for VL treatment it has to be ensured that they are used for the purpose of sLAB treatment only. Preparation and administration of ambisome requires skilled human resources. Even though side effects and previously observed serious allergic reactions may be rare and even though MSF reported that the LAB (triple dose) treatment was managed properly by primary health care workers in rural Bangladesh, health staff should be able to manage potential adverse reactions such as drop of haemoglobin, allergic reactions, and drop in blood pressure.

## 5. Conclusion

Comparing favouring and limiting factors, the enabling factors clearly outweigh the limiting factors for sLAB treatment in Bangladesh. As the efficacy trial under field conditions has shown favourable results [19] the sLAB treatment appears to be the most promising option for VL treatment in Bangladesh.

## Supplementary Material

The primary data collection tools of the structured questionnaires for patients, topic guide for focus group discussions and open interviews can be cosidered in supplementary material.

## Figures and Tables

**Table 1:** Items needed for IV application of LAB and their availability in 5 rural hospitals (UHCs) of Bangladesh.

Items for preparation of ambisome treatment: availability	Item available at UHCs *n*/*N*
5% DA (500 mL)	
No continuous supply	3/5
Not available	2/5
Distilled water 10 mL	0/5
Distilled water 5 mL	
No continuous supply	3/5
Not available	2/5
5% DA (500 mL)	
No continuous supply	3/5
Not available	2/5
Disposable infusion sets	0/5
Disposable syringe	
10 mL available	1/5
20 mL available	0/5
Disposable syringe 5 mL	
Continuous supply	3/5
No continuous supply	2/5
Scissors available	4/5

Items for administration of ambisome	
Gloves	
Continuous	2/5
Not continuous	2/5
No	1/5
Cotton balls available	5/5
Chlorhexidine bottle (70%)	2/5
IV canulla 18 g	
Continuous supply	3/5
No continuous supply	2/5
IV canulla 24 g	1/5
IV canulla 22 g	1/5
IV canulla 20 g	0/5
Micropore available	5/5

**Table 2:** Drugs needed to treat adverse events of sLAB treatment in 5 rural hospitals (UHCs).

Items for treatment of adverse events: availability	Item available at UHCs *n*/*N*
Prophylactic treatment before administration of sLAB	
Paracetamol (tablets) available	5/5
Antihistaminic tablets available	5/5
First aid drugs	
Nebulizer available	5/5
Adrenalin available	2/5
Chlorpheniramine available	5/5
Dexamethasone	
Available	3/5
No continuous supply	2/5
First aid drugs are in one place	0/5

**Table 3:** Patients' experiences and perceived benefits by the sLAB and miltefosine treatment regimen.

Indicator	sLAB *N* = 299	Miltefosine *N* = 22	*P* value
Resolution of fever in days, mean (SD)	2.48 (0.86)	17.58 (11.34)	<0.001
Nausea/vomiting after treatment % (*n*)	1.3 (4)	86.4 (19)	<0.001
Diarrhoea % (*n*)	0.00 (0)	63.6 (14)	<0.001
Abdominal pain % (*n*)	0.7 (2)	27.3 (6)	<0.001
Mean of time to recovery in days (SD)	2.39 (0.731)	17.36 (12.385)	<0.001
Mean of time in days to start working after treatment (SD)	2.72 (1.819)	52.82 (33.987)	<0.001
Income loss due to recovery time	2.72 days × 0.30$^1^/day = 0.816$	52.82 days × 0.30$^1^/day = 15.846$	<0.001
Income loss (working days lost when receiving treatment)	2.39 days × 0.3$/d = 0.72$	17.36 days × 0.3$/d = 5.2$	<0.001

^1^Average daily income per person in a VL affected household in Bangladesh, calculated by Anoopa Sharma et al. 2006 [10].

## List of Abbreviations

FGD: Focus group discussion
MSF: Médecins Sans Frontiéres
UHC: Upazila Health Complex
UH&FPO: Upazila Health and Family Planning Officier
US-FDA: United States Food and Drug Administration
sLAB: Single dose liposomal amphotericin B
SOP: Standard operation procedure
VL: Visceral leishmaniasis
WHO: World Health Organization
TDR-WHO: Special Programme for Research and Training in Tropical Disease at the World Health Organization.
