# Supplementary material for: Introducing Single Dose Liposomal Amphotericin B for the Treatment of Visceral Leishmaniasis in Rural Bangladesh: Feasibility and Acceptance to Patients and Health Staff
Source: J Trop Med. 2014 Jan 21;2014:676817. doi: 10.1155/2014/676817 (PMC3918353; doi:10.1155/2014/676817)
Supplement: Supplementary file 1 — The primary data collection tools of the structured questionnaires for patients, topic guide for focus group discussions and open interviews can be cosidered in supplementary material. [file 676817.f1.pdf]

International Centre for Diarrheal Disease Research, Bangladesh

Introducing single dose liposomal Amphotericine B for the treatment of visceral leishmaniasis in rural Bangladesh: feasibility and acceptance to patients and health staff

Principal Investigator: Dr. Dinesh Mondal

Focus Group Discussion

Before starting the discussion:

- Introduction of the researchers, the process of the interview and signing the consent form
- Short introduction of everybody: Name, Gender, Age, Living area, Job, Husband's Job, House type, Religion, Tribe

Topic guide:

1. When had you been treated for kala-azar and for how many times in your life?
2. What where the symptoms you experienced and what did you do?
   (Prompts: Health inspector, traditional healer, village doctor (Quake), UHC, Pharmacy,)
3. What financial effort did you have to make?
   (Prompts: transport costs, pay to get into UHC, doctor chamber, loose daily wages)
4. *Individually*:
   a. How much did you have to pay in total for the treatment
   b. Did you get any incentives for your treatment?
   (transport, admission fee, doctors fee, daily wages lost)
5. What kind of treatment did you have to take and how did you have to take it?
   (Prompts: daily, for 30 days, tablets)
6. Did you receive complete treatment?
7. Did it cause any side effects and in what moment of treatment?
   (Prompts: a) kind: (nausea, vomiting, diarrhoea, fever, jaundice, weakness),
   b) moment: always after treatment, sometimes after taking medication, only the first time, just once)
8. Where do you see difficulties for you concerning this treatment?
   (Prompts: intake (28 days tablets), side effects, costs (transport, doctor), coming for check-up 3 times)
9. How many times did you have to come for check-up and could you manage it?
   (Prompts: every week check-up, no time, too expensive, no need, good for monitoring)
10. What did you like about the treatment?
    (Prompts: time to recovery, form of intake, free of costs)
11. *Individually*: If you had different options, which way of treatment would you choose?
    a. 1 infusion and one night in hospital,
    b. Tablets for 28 days including 4 visits to the hospital (one visit every week),
    c. 1 infusion and then 10 Injections (i.m.) staying for 10 days at the hospital
    d. 1 injection, 1 night in the hospital and then oral treatment for 10 days
    e. 10 Injections (i.m.) and oral treatment for 10 days staying 10 in hospital

12. *Individually:* Would you recommend this treatment to other family members?

International Centre for Diarrheal Disease Research, Bangladesh

Study title: Study Proof of concept to determine the feasibility of single dose liposomal Amphotericin B for Visceral Leishmaniasis at the Upazila level in Bangladesh

Principal Investigator: Dr. Dinesh Mondal

Interview with UHFPO:

1. How many patient beds do you have ? _______________
2. Usual hospital bed occupancy (%/month)_______________________________
3. How many kala-azar patients do you treat each month?__________________
4. Could they be treated here if they would have to stay 1 night?______________
5. Number of nurses in total and per day____________, _____________
6. Number of nurses in the ward in charge of kala-azar patients ______________
7. Nurses trained for AmBisome (preparation and application) ________________
    a. Date of the training: ______________
    b. Refreshment needed? (nurses) ______
8. Number of kala-azar doctors?____
9. Are these doctors trained in AmBisome treatment?______________________
10. How long is the duration of an interruption of the power supply?___________
11. How many times/month it happens for >2h?_____________________________
    ____
12. Strategy in times of load shedding_____________________________________
    ____
13. Capacity to mesure HB/Hemocue?__________________________________
14. What is your opinion about feasibility of AmBisome treatment at you UHC?
    ____

____

2. Checklist of supplies for facilities

1. Assessment at EPI-ROOM

**Storage capacity**	
Refrigerators:<br>a) number and type of working refrigerators (-80°C, -20 °C, 4-8°C, 4-25°C)<br>b) number and type of refrigerators switched off (-80°C, -20 °C, 4-8°C, 4-	

25°C)		
Generator/ alternative power supplies		
How many litres of fuel do you get each month?		
How much do you need in fact?		
Is it available close to the UHC?		
**2. general ward**		
**Measurement supplies**	**Requirements**	**Amount available**
BP machine -Adult	1	
Stethoscope-Adult	1	
BP machine -Child	1	
Stethoscope- Child	1	
Measuring tape	1	
Thermometer	2	
Torch light	1	
Temperature measuring alarm	1	
Weight machine	1	
Height machine		
**Drug storage capacity**		
Refrigerator (-80°C, -20 °C, 4-8°C, 4-25°C)		
Generator/ alternative power supplies		
Ice packs		
Cabinet / Almirah	1	
File Cabinet		
**Treatment supplies**		
5% DA (500ml)	Available?<br>Continuous supply?	
Distilled water 10 ml	Available?<br>Continuous supply?	
Distilled water 5 ml	Available?<br>Continuous supply?	
Tab. Paracetamol	Available?<br>Continuous supply?	

Tab. Chlorpheniramine maleate	Available?<br>Continuous supply?	
Alternative:		
Disposable syringe 10ml	Available?<br>Continuous supply?	
Disposable syringe 20 ml	Available?<br>Continuous supply?	
Disposable syringe 5ml	Available?<br>Continuous supply?	
Cotton ball	Available?<br>Continuous supply?	
Chlorhexidine bottle (70%)	Available?<br>Continuous supply?	
disposable infusion sets	Available?<br>Continuous supply?	
Gloves	Available?<br>Continuous supply?	
Lancet	Available?<br>Continuous supply?	
IV canula 18g	Available?<br>Continuous supply?	
IV canula 22g	Available?<br>Continuous supply?	
IV canula 20g	Available?<br>Continuous supply?	
Micropore 2inch	Available?<br>Continuous supply?	
Micropore 1 inch	Available?<br>Continuous supply?	
Paracetamol suppository 125mg	Available?<br>Continuous supply?	
Paracetamol suppository 250mg	Available?<br>Continuous supply?	
Paracetamol suppository 500mg	Available?<br>Continuous supply?	
Scissors	2 pairs	
First Aid kit:		
Nebulizer	1	
Adrenalin	Available?<br>Continuous supply?	
Antihistaminic (Chlorpheniramine) tabl.	Available?<br>Continuous supply?	
Dexamethasone	Available?<br>Continuous supply?	
Are those drugs in one place?	yes	

Nurses: Time capacity to treat patients<br>(1 h/pt)		
Nurses: Are you trained?		
**3. pathology department:**		
rk 39 strip test	Available?<br>Continuous supply?	
Pregnancy test strip	Available?<br>Continuous supply?	
Capacity to mesure HB?		

A. INTERVIEWS WITH DOCTORS: EXPERIENCES WITH KALA-AZAR TREATMENT

Place of interview:……………………….,

☐ Female ☐ Male Age……….

Profession:…………………. Place of work: …………………………………..

1. How long have you been working with KA patients?
2. How many patients have you treated during this time?
3. How do you diagnose a patient suspected to have Kala-Azar?
4. What other laboratory testing do you do before starting a treatment?
5. How frequently do you do a pregnancy test in women in fertile age?
6. What type of drugs you have been using to treat KA patients?
7. Which of these do you use now?
8. What do you think is the best option for treatment of a KA patient?
9. Have you heard about LAB?
10. What do you think about it?
11. What kind of side effects do you see treating with:
    a. Miltefosin
    b. AmBisome
    c. Paromomycin
    d. SSG
    e. SDA
12. Which difficulties do you see using LAB?
13. What side effects would you expect using LAB?
14. Do you think the health staff will be able to handle the additional work?
15. Do you store and provide EPI vaccines?
16. How do you store them?
17. What kind of strategies do you have for power cuts?
18. Do you think the cold chain can be accomplished storing LAB at 4°C- 25°C?
19. Do you think treatment with LAB would be possible?
20. What is your overall opinion about the feasibility of AmBisome treatment in the routine programme?
21. Do you have any training on AmBisome management?
22. Do you havereceived the Standard Operating Procedures (transportation of AmBisome; dilution : allergic test and its infusion)?

QUESTIONNAIRES FOR PATIENTS WITH AMBISOME TREATMENT

A. <u>EXIT INTERVIEW DIRECTLY AFTER TREATMENT</u>

(To be applied in Upazila Health Complex where Single Dose Ambisome is given compared to UHCs where other treatment options are provided)

Place of interview……………………………………………………….

Patient ID……………………………………………………..

Kala-azar treatment with: 1. Ambisome Single Dose 2. Miltefosine 3. SAG (Antimonials) 4. Paromycin 5. Combinations; 6. AmBisome (multiple doses)

☐ Female ☐ Male Age……….

1. What is the name of the disease you have been treated for………………….(1. Correct 2.incorrect)
2. What causes that disease (called Kala-azar)'? 1. Microbes/parasites 2. Sand flies 3. Malnutrition 4. Poor water 5. Heat, cold, too much work 6. Other
3. What did the doctor explain about the treatment:? (*tick correct answers*)
   1. For how long is the treatment ………
   2. Side effects of the treatment ……
   3. Possible dangers of the treatment?.....
   4. Advice on nutrition (There is no mention at all about it anywhere in the ICF)
   5. Any other advice, explain……………………………………………………..
4. Did the treatment cause anything of the following:

   O Pain O Fever O Nausea, vomiting
   O Diarrhoea O Back pain O Joint pain
   O Anything else?...................... O Nothing was wrong
5. If your brother or sister would have the same disease, would you recommend them the same type of treatment?
   1. Yes , Because…………………………………………………..
   2. No , Because……………………………………………………
6. What would you prefer as a treatment? (*tick one of the following answers*)

   O One visit, one infusion and one night in hospital,
   O Tablets for 28 days including 4 visits to the hospital (one visit every week),
   O Injections for 10-30 days
7. What did you like about the treatment you just received?..................................
8. What did you dislike about the treatment?..................................
9. What could be done better?...............................
10. Do you have family coming with you? 1. Yes, 2. no
11. Did they have to stay at the hospital? 1.Yes, 2. no
12. Did they have compensate for their stay and how much?
13. What would be the normal costs to come to the hospital for this kind of treatment?
14. How many days of work do you loose coming here ?

B. EXIT INTERVIEW: ONE MONTH AFTER TREATMENT

Place of interview…………………………………………………………..
Patient ID…………………………………………………….

Kala-azar treatment with: 1. Ambisome Single Dose 2. Miltefosine 3. SAG (Antimonials) 4. Paromycin 5. Combinations; 6. AmBisome (multiple doses)

☐ Female ☐ Male Age………..

1. How do you feel today?
   O Excellent O Fine O More or less well O Sick O Very sick
2. During the time since your treatment, did you feel anything of the following?

**Complaints**	**How many weeks after treatment?**	**For how many days?**
Fever		
Nausea, vomiting		
Diarrhoea		
Abdominal pain (local word)		
Joint pain		
Back pain		
Anything else		
Nothing was wrong	-	-

3. After how many days did you feel better?.......................................
4. After how many days could you start working, go to school or start your daily activities again?...
5. If your brother or sister would have the same disease, would you recommend them the same type of treatment?
   1. Yes, Because…………………………………………………..
   2. No, Because……………………………………………………
6. What would you prefer as a treatment? (*tick one of the following answers*)
   O One visit, one infusion and one night in hospital,
   O Treatment for 28 days including 4 visits at the hospital (one every week),
   O Injections for 10-30 days
7. What did you like about the treatment you just received?..........
8. What did you dislike about the treatment?...........
9. What could be done better?...............................
10. Do you have family coming with you? 1. Yes, 2. no
11. Did they have to stay at the hospital? 1. Yes, 2. no
12. Did they have to pay for their stay and how much?
13. What are the costs for you to come to the hospital?

QUESTIONNAIRES FOR PATIENTS WITH AMBISOME TREATMENT OR OTHER VL TREATMENTS

A. <u>INTERVIEW AFTER TREATMENT</u>

(to be applied to patients treated with Miltefosine)

Place of interview…………………………………………Patient ID……………………………………………………..

Kala-azar treatment with: 1. SAG (Antimonials) 2. Miltefosine ; 3. AmBisome single dose (10 mg); 4.Paromycin; 5. Miltefosin (10days) + AmBisome single dose (5mg); 6. AmBisome triple dose (15mg); 7. AmBisome single dose + Miltefosin (10 days); 8. Miltefosin (10days) + Paromomycin (10days);

☐ Female ☐ Male Age………. Date of illness:…………………………………………..

1. What is the name of the disease you have been treated for…………………..(1. Correct 2.incorrect)
2. When have you been treated ....at ....
3. Where did you do the rK39 test?
4. How much was the test?
5. How many times in your life? ....
6. What causes that disease (called Kala-azar)'? 1. Microbes/parasites 2. Sand flies 3. Malnutrition 4. Poor water 5. Heat, cold, too much work 6. Other
7. What did the doctor explain about the treatment:? (*tick correct answers*)
   1. For how long is the treatment ………
   2. Side effects of the treatment ……
   3. Possible dangers of the treatment?.....
   4. Advice on nutrition …………………………..
   5. Any other advice, explain………………………………………………………..
8. To females (15-45 years of age):
   a. Did you do a pregnancy test before starting the treatment? …………….
   b. Did the doctor explain anything in terms of pregnancy or child wish?...
9. During the time since your treatment, did you feel anything of the following?

**Complaints**	**How many weeks after treatment?**	**For how many days?**
Fever		
Nausea, vomiting		
Diarrhoea		
Abdominal pain (local word)		
Joint pain		
Back pain		
Anything else		
Nothing was wrong	-	-

10. Did it happen that you could not take your treatment?
    a. Why………………………………………………………………

11. After how many days did you feel better?..
12. After how many days could you start working, go to school or start your daily activities again?...
13. If your brother or sister would have the same disease, would you recommend them the same type of treatment?   1. Yes , Because…………………………………………………..
    2. No , Because…………………………………………………

14. What did you like about the treatment you just received?….................................
15. What did you dislike about the treatment?....................................
16. What would you prefer as a treatment if you could choose one of the following?

    O 1 infusion and one night in hospital,
    O Tablets for 28 days including 4 visits to the hospital (one visit every week),
    O 1 Infusion and then 10 Injections (i.m.) for 10 days at the hospital
    O 1x Injection, 1 night in the hospital and then oral treatment for 10 days
    O Injections (i.m.) and oral treatment for 10 days staying 10 in hospital

17. How many times did you have to come for check up?
18. Could you manage to come always? 1. Yes, 2. No, because…………………………………
19. Did you have to stay at the hospital at any time? 1.Yes, 2. no
20. Do you have family coming with you to the hospital? 1. Yes, 2. no
21. What would be the normal costs to come to the hospital for this kind of treatment each time?
22. How many days of work did you lose going to the hospital?
